# Input data quality control for NDNQI national comparative statistics and quarterly reports: a contrast of three robust scale estimators for multiple outlier detection

**DOI:** 10.1186/1756-0500-5-456

**Published:** 2012-08-25

**Authors:** Qingjiang Hou, Brandon Crosser, Jonathan D Mahnken, Byron J Gajewski, Nancy Dunton

**Affiliations:** 1 Department of Biostatistics, University of Kansas Medical Center, 3901 Rainbow Blvd, Kansas City, KS 66160, USA; 2 School of Nursing, University of Kansas Medical Center, 3901 Rainbow Blvd., Kansas City, KS 66160, USA

**Keywords:** NDNQI, Interquartile range, Median absolute deviation, FAST-MCD, Outlier, Quality control

## Abstract

**Background:**

To evaluate institutional nursing care performance in the context of national comparative statistics (benchmarks), approximately one in every three major healthcare institutions (over 1,800 hospitals) across the United States, have joined the National Database for Nursing Quality Indicators® (NDNQI®). With over 18,000 hospital units contributing data for nearly 200 quantitative measures at present, a reliable and efficient input data screening for all quantitative measures for data quality control is critical to the integrity, validity, and on-time delivery of NDNQI reports.

**Methods:**

With Monte Carlo simulation and quantitative NDNQI indicator examples, we compared two ad-hoc methods using robust scale estimators, Inter Quartile Range (IQR) and Median Absolute Deviation from the Median (MAD), to the classic, theoretically-based Minimum Covariance Determinant (FAST-MCD) approach, for initial univariate outlier detection.

**Results:**

While the theoretically based FAST-MCD used in one dimension can be sensitive and is better suited for identifying groups of outliers because of its high breakdown point, the ad-hoc IQR and MAD approaches are fast, easy to implement, and could be more robust and efficient, depending on the distributional property of the underlying measure of interest.

**Conclusion:**

With highly skewed distributions for most NDNQI indicators within a short data screen window, the FAST-MCD approach, when used in one dimensional raw data setting, could overestimate the false alarm rates for potential outliers than the IQR and MAD with the same pre-set of critical value, thus, overburden data quality control at both the data entry and administrative ends in our setting.

## Background

To establish the benchmark and monitor nursing sensitive quality indicators across the United States, the American Nurses Association (ANA) established the National Database for Nursing Quality Indicators® (NDNQI®) in 1998 [1]. With over 1,800 hospitals at present, NDNQI collect unit-level data online through a secured database and provides each member institution quarterly report with 8-quarter trend data, along with national comparative statistics stratified by hospital staffed bed size, teaching or Magnet status, unit type, and various other characteristics of institutional preference. With a dynamic input from over 18,000 hospital units, NDNQI compiles over 200 quantitative measures of nursing care structure, process, and outcomes. For input data quality control, NDNQI conducts one dimensional data quality check for various quantitative measures at first, potential outliers are flagged at the univariate level for correction or confirmation to ensure the quality and overall validity of national comparative statistics by various stratifications. Detecting and evaluating valid extreme observations, on the other hand, may be just as important to participating hospitals since they identify what needs to be exemplified or improved to better their services. Besides multilevel validation rules and compatibility checks with online data entry through the secured NDNQI database, an interactive statistical data screening procedure with up to three rounds of overnight univariate data screening for potential outliers has been implemented since the beginning of NDNQI. The statistical data screening starts immediately once a quarterly data entry deadline is approached and continues until all questionable inputs are resolved or confirmed through the hospital site coordinator, the institution’s designated data manager. At present, we rely on the theoretically based FAST-MCD approach [2], because it’s readily available with most commercial statistical packages and it is applicable to one dimensional outlier detections with high breakdown point property. With the continuous growth of NDNQI in both number of facilities and new quantitative measures, we need to expand the initial statistical screening on input data and run a most efficient and reliable quality control to ensure the on-time delivery of high quality quarterly report, one of the most frequent suggestions on the 2008 NDNQI customer satisfaction survey [3]. Currently, NDNQI quarterly report uses Bayesian hierarchical modeling [4] and Box-Cox transformation approach [5] for hospital report cards and NDNQI national comparative statistics once the institutional data are deemed clean or reconfirmed after initial raw data screening. Robust regression methods with multivariate outlier detection techniques are also available and have been intensively reported in literature [6-10], though we focus this work on univariate outlier detection as guided by our application for NDNQI processes.

Outliers refer to abnormal observations that do not conform to the pattern (model)suggested by the majority of the cases in a data set [11], which can result from different reasons. Some of them reflect unit-level superior/deficient performance in measured quality, as in the case for NDNQI, but are true observed values; others may be derivatives of miscalculation, wrong definition or simply typos. Many methods are available for outlier detection [2,12-19], and most of them are distance-based on one kind or another robust measure of location and scatter (scale estimator) [2,17,20-22]. Detection and examination of potential outliers are integral parts of data analysis [23-25], because the presence of outliers may alter statistics, reduce the power of a test, and even lead to incorrect conclusions. On the other hand, outliers are often of primary interest in searching for superiority, such as in biological breeding, geological exploration, and pharmaceutical research. In NDNQI, an outlier for a certain indicator could signal an outstanding performance or inadequate service in nursing care, supply, and/or skill [26], which in turn could provide critical feedback to the hospital administration. Comparisons of different methods for detecting outliers have also been well reported by Kianifard and Swallow [27], Hadi and Simonoff [28], Serbert et al. [29], and most recently, Billor and Kiral [11]. Most previous works focused on residuals from a regression model in which the residuals are roughly normally distributed for the bulk of observations. The primary interest for this study, however, is to investigate the extent to which the detection capability and robustness of three different approaches, based on FAST-MCD, IQR, and MAD, will be affected if the majority of the underlying population deviates from the normal assumption. This is because a) most NDNQI indicators have skewed distributions, b) factors with structural effect are potentially large, unknown, and most likely differ from indicator to indicator, and c) we emphasize on checking the validity of the raw input data.

Among the commonly used methods, the FAST-MCD approach is most popular because it is robust, sensitive, and applicable to both univariate and multivariate outliers. The FAST-MCD approach is based on the iterative estimates of multivariate location (T) and scatter (C) obtained from *h* observations (out of a total of *n*) whose covariance has the lowest determinant, with *h*≥(*n* + *p* + 1)/2, and *p* representing the dimension of the data. In the extreme case, the robust estimates of location and scatter could be based on the simple majority (*n*/2 +1) of all observations. Once the scatter C and location T are determined, they are used in the following equation, in matrix notation, for calculating the robust distance (**D**) for all *n* data points:

(1) $$D=\sqrt{{\left(X-T\right)}^{T}C^{-1}\left(X-T\right)}$$

where, the squared distance is Chi-square distributed, $D^{2}\sim \chi_{p}^{2}$, with *p* representing the dimension in column of the X matrix. The outlyingness of an observation is assessed by its distance (**D**) from location T of (1) compared to the square root of a critical value of the $\chi_{p}^{2}$ distribution [30]. The distance is robust because all (*n - h*) observations that did not contribute to the covariance matrix with the lowest determinant have zero weight on T and C, and thus have no effect on the measure of D. Consequently, the robust distances for all *n* observations are not affected by the number (if less than (*n* + *p* + 1)/2) and magnitude of potential outliers. If a large proportion of the data are concentrated at a single lower end point, FAST-MCD approach is more likely to fail because robust distance can not be calculated due to C being zero. It is also possible that the remaining (*n* - *h*) subset be all declared outliers if they tend to be isolated in groups but not necessarily separated by large distances from the h observations. As a result, the FAST-MCD approach could mislead depending on the nature of the data distribution. In this paper, we focus on detecting outliers in the raw (also called pre-aggregated) data. The FAST-MCD, used in one dimensional setting, along with the other two approaches, serves as a benchmark for comparison, because the theoretically based MCD approach is sensitive to groups of outliers with high breakdown point. Thus, T, C, D, and the X _(N×P)_ in matrix notation under multivariate framework are reduced to scalars for point estimates of T, C, D, and X _(N×1)_, respectively, as in the one dimensional cases.

Besides the FAST-MCD, two well-known and easily computed robust measures of scatter, the Inter Quartile Range (IQR) and Median Absolute Deviation from the median (MAD), were reported to be effective for detecting multiple outliers [17]. They are defined as:

(2) $$\text{IQR}=75^{\text{th}}\text{percentile}-25^{\text{th}}\text{percentile}$$

and

(3) $$\text{MAD}=\text{median}\left\{\left|x_{i}-\text{median}\left(x_{i}\right)\right|\right\}$$

where, $x_{i}$ represents all observations with *i* ranges from 1 to *n*.

Through simulation study on residuals from a regression model $y_{i}=x_{i}+{ɛ}_{i}$, where $x_{i}$and ${ɛ}_{i}$are generated as uniform *U*(0, 15) and standard normal *N*(0, 1) random variables, Swallow and Kianifard [17] showed both IQR and MAD asymptotically approach the standardized variance of 1.00 for $\varepsilon_{i}$ through constant divisors of 1.369, 1.363, 1.355 and 0.639, 0.658, 0.666 with sample sizes of 25, 50 and 100, respectively. They suggested adjusting IQR or MAD through one of the constant divisors as robust estimates ( $\hat{\sigma }$) of σ for testing the null hypothesis that an observation is an outlier if *e*_*i*_ / $\hat{\sigma }$ is greater than or equal to a preselected critical value for standard normal distribution *N*(0, 1) (1.96 for 5% or 2.54 for 1% significance level). They proposed a stepwise strategy for testing the null hypothesis that the *j*^*th*^ ( *j* = *p* + 1, . . . , *n* ) observation is not an outlier. After fitting the regression model, the first *p* observations with the smallest absolute value of studentized residuals were used for computing the *n - p* recursive residuals ( $w_{j}$) as defined by Brown, Durbin, and Evens [31]. The largest of the test statistics | $w_{j}$/ $\hat{\sigma }$| is compared to a critical value, and the no-outliers hypothesis is rejected when the test statistic is greater or equal to the pre-selected critical value. The procedure is repeated by removing the observation from computation until the no-outliers hypothesis cannot be rejected. Swallow and Kianifard concluded that using ordinary least square residuals, studentized residuals, or the recursive residuals has little effect on the critical values for testing no-outliers hypothesis at 0.1, 0.05, or 0.01 significance levels with either IQR or MAD as scale estimates. We chose IQR/1.355 or MAD/0.666 as the robust estimate of scale since both simulation and NDNQI example data used in this study are substantially large.

## Methods

The cleaned NDNQI 3^rd^ quarter data in 2007 was used to explore the distributional property of indicators and how data distribution affect robustness and false alarm rate by the three scale estimators. The study was approved by the IRB of the Human Subjects Committee at The Kansas University Medical Center. A total of 12,145 units contributed, at least partially, to the NDNQI database for the 3^rd^ quarter in 2007. Based on the extract, 146 quantitative measures were computed for constructing nursing sensitive quality indicators at hospital-unit level [32]. Among all indicators, we selected Total Falls Per 1,000 Patient Days, Injury Falls Per 1,000 Patient Days, Percent of PIV Sites with Vesicant Solution, Percent of Surveyed Patients with Pressure Ulcers, and Average Number of Pain Assessments per Patient Initiated in 24 Hours, because these measures represent the wide range of data distributions among all indicators. For each of the 7 selected measures, the critical value with FAST-MCD was set at 5.02 for the squared robust distance, corresponding to 2.5% significance level for χ^2^ distribution with 1 degree of freedom. The critical value for the IQR and MAD approaches was 2.24, corresponding to the 1.25% lower and upper percentiles for two-sided test with the standard normal distribution. In each case, around 2.5% of the observations were targeted for recheck. We thought it was necessary to keep the critical value at 2.5% level considering NDNQI commitment to data integrity and quality, the dimension of data to be screened, the number of hospitals involved, and the available data management resources.

A close look of all indicators revealed that their distributions are highly skewed to the right, and a Gamma distribution with different shape and scale parameters would provide each the best goodness of fit. Therefore, we performed a simulation study by generating Gamma random variables *X* ~ Г(*α*, *β*), using SAS® RANGAM [33] function with various scale (*β*) and shape (*α*) parameters. The pairs of *β* and *α* were selected such that the skewness $\left(y,=,\frac{2}{\sqrt{\alpha }}\right)$ of *X* ranged from around 0 (close to normal) to 4 (heavily skewed to the right), but the means of *X*$\left(X\left(\mu =\alpha \times \beta \right)\right)$ remained the same. SAS MCD CALL routine was used for calculating the robust distance, while the inter quartile range in (2) and median absolute deviation from the median in (3), along with the skewness and other descriptive statistics were obtained with the SAS UNIVARIATE procedure. A SAS macro program was written to identify potential outliers and to combine and compare results with the three methods.

To contrast the ability to identify true outliers by each method, we adjusted the Monte Carlo simulation such that 10 observations (1%) were planted at random as known outliers in each generated data set along with the remaining 990 data points (99%) at various level of asymmetry as described above.

For real case application, we computed a few NDNQI indicators both before and after data cleaning, using 2007 NDNQI 4^th^ quarter data, and then checked each indicator for potential outliers to compare the sensitivity and efficiency of the three approaches.

## Results

### NDNQI quarterly report data in 2007

If FAST-MCD, IQR, and MAD approaches were equally robust and efficient for NDNQI 2007 3^rd^ quarter data, we should expect around 2.5% of reporting units for each indicator to be identified for recheck or validation by hospital site coordinators. In this case, the false alarm rate was 2.5% since all questionable observations were rechecked and deemed as clean. Unfortunately, all three methods overestimated the target for Total Falls per 1,000 Patient Days, but their differences were within 2%when the indicator’s distribution was neither too skewed (γ = 1.772) nor too concentrated at the lower end (Table 1). The rate of overestimation went higher with the increase in skewness, especially with the FAST-MCD approach, as shown by the Injury Falls per 1,000 Patient Days, Percent of Surveyed Patients with Hospital Acquired Pressure Ulcers, and Total Nursing Hours per Patient Day. As the data skewed more to the right, such as Percent of PIV Sites with Vesicant Solution, FAST-MCD classified over 10% more units into the potential outlier category, compared to IQR and MAD methods. In an extreme case, the inflated false alarm rate by the FAST-MCD approach reached as high as 20% for Percent of Registered Nurses, and up to 30% for Percent of Surveyed Patients with Hospital Acquired Pressure Ulcers, compared to those identified by the IQR and MAD methods. Among the three approaches, the IQR was most consistent in terms of maintaining the preset 2.5% false alarm target across a wide range of asymmetry in data distribution followed by the MAD approach while the data is not heavily skewed (γ < 3). Furthermore, both MAD and FAST-MCD approaches are susceptible to failure when the data heavily concentrate at the lower end of the distribution even if the skewness is relatively low (γ < 2), as observed with NDNQI Prior Risk Assessment for Pressure Ulcer, Total Nursing Hours Per Patient Day, Assisted Patient Falls Rate, and Multiple Site PIVs. In such cases, neither FAST-MCD nor MAD will be able to estimate the scale, thus fail to pick any observation as potential outlier.

**Table 1:** Distributional skewness and false alarm rates for potential outlier check by IQR, MAD, and FAST-MCD approaches for selected NDNQI indicators

**NDNQI indicators**	**Skewness** **(γ)**	**False alarm rates by different approach**			
		**Target**	**IQR**	**MAD**	**FAST-MCD**
Total Falls Per 1,000 Patient Days	1.772	2.5%	3.97%	3.50%	5.55%
Total Injury Falls Per 1,000 Patient Days	3.064	2.5%	4.15%	4.88%	28.34%
Percent of Total Nursing Hours Provided by RNs	2.412	2.5%	5.58%	7.25%	30.34%
Total Hospital Acquired Pressure Ulcer	3.608	2.5%	6.61%	10.24%	49.04%
Total Number of Ulcers	2.456	2.5%	3.49%	2.80%	16.69%
Average Pain Assessments in 24 Hours	1.622	2.5%	8.08%	7.38%	8.78%
Prior Risk Assessment for Pressure Ulcers	−1.675	2.5%	14.97%	-*	48.01%
Total Nursing Hours per Patient Day	1.533	2.5%	6.02%	9.22%	21.57%
Percent Vesicant PIV	1.172	2.5%	7.44%	-	-
Assisted Falls	1.267	2.5%	7.76%	-	-
Assault Rate	4.568	2.5%	9.23%	12.62%	34.46%
PIV-Multiple Sites	3.828	2.5%	-	-	-

* denote the method failed and led to missing values for the statistic.

**Figure 1:**
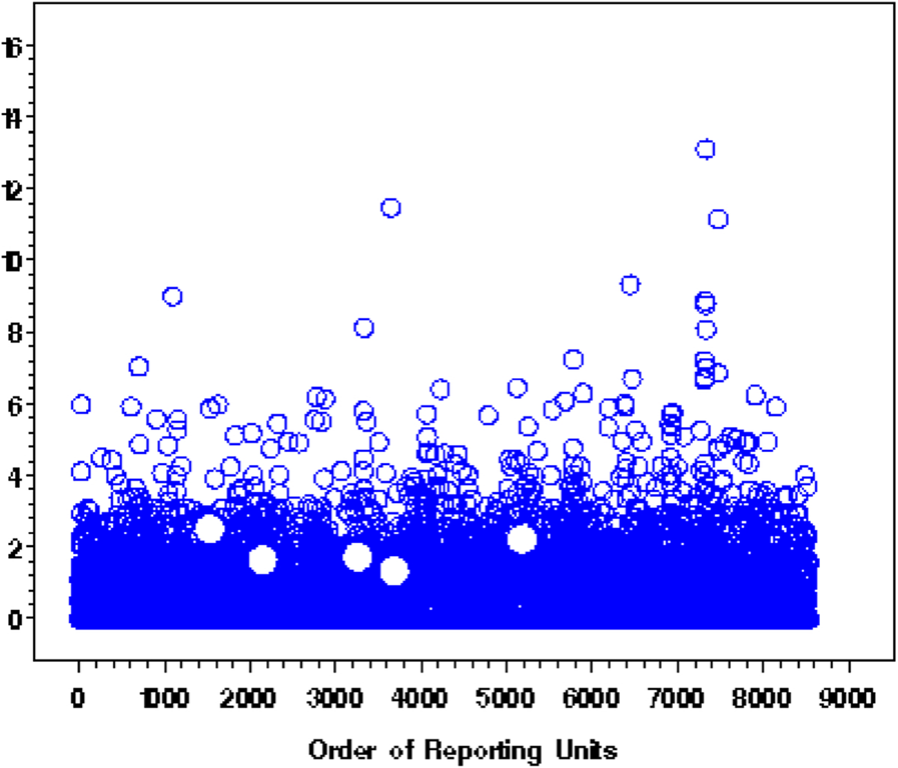
**With NDNQI Injury Fall Rate for 2007 4**^**th**^** Quarter, the 5 flagged units for rechecking with FAST-MCD approach are all false alarms, which the IQR and MAD approach did not flag at first.**

After the NDNQI open data entry period was closed for 2007 4^th^ quarter, RN Hours Per Patient Day, Total Falls Per 1,000 Patient Days, and Injury Falls Per 1,000 Patient Days (Table 2) were chosen as example for checking potential outliers at the 2.5% targeted significance level. Despite a considerably larger percentage of reporting units were flagged for each indicator by all three methods, most of the flagged reporting units were confirmed as true values (false outliers) by the corresponding hospital site coordinators after rechecking. All three methods were able to pick up nearly the same set of observations as true outliers (equally sensitive), which were corrected by site coordinators in the cleaned database (Table 2). A few more outliers picked by FAST-MCD in Table 2 may be attributed to the higher percentage of false outliers (identified for recheck but reconfirmed as true values) than it’s robustness and sensitivity. As Figure 1 illustrated, the five reporting units picked up by FAST-MCD but ignored by IQR and MAD for Injury Falls Per 1,000 Patient Days are not significantly different from the bulk of the remaining units. The percentage of false outliers by FAST-MCD, however, is considerably larger than those given by IQR or MAD, suggesting more time and effort could be saved on data cleaning both at the hospital input and NDNQI administrative ends by using IQR or MAD approaches.

**Table 2:** Total number of units reporting with data, required for reconfirm after screening, with outliers corrected, and false alarm rate by different approach

**RN hours per patient day by unit type**			**IQR**			**MAD**			**FAST-MCD**		
	***N***_**0**_	***N***_**1**_	***N***_**2**_	***N***_**3**_	***Post***	***N***_**2**_	***N***_**3**_	***Post***	***N***_**2**_	***N***_**3**_	***Post***
Critical Care	1940	55	110	8	5.45%	109	8	5.30%	158	11	8.81%
Step Down	1259	22	60	2	4.69%	54	1	4.36%	87	2	7.37%
Other	4895	119	189	18	3.78%	179	17	3.68%	246	21	5.47%
Rehabilitation	451	8	18	0	3.99%	16	0	3.55%	16	0	3.95%
Neonatal	366	11	38	5	10.1%	38	5	10.1%	44	5	13.3%
Pediatric Critical Care	152	5	7	1	5.26%	7	1	5.26%	7	1	5.88%
Pediatric Step Down	33	1	6	0	18.2%	6	0	18.2%	6	0	25.8%
Pediatric Medical	99	3	5	0	5.05%	10	1	9.09%	19	1	20.7%
Pediatric Surgical	37	2	3	1	5.41%	3	1	5.41%	4	1	8.33%
Psychology ChildAd	373	11	24	2	7.69%	22	2	7.69%	26	1	9.91%
Psychology Gerip	117	4	10	1	5.15%	10	1	5.15%	11	1	10.1%
**Falls Indicators**											
Fall Rate	8555	25	290	1	3.42%	286	1	3.37%	479	2	6.18%
Injury Fall Rate	8555	10	300	0	3.50%	397	0	4.62%	2249	5	28.9%
Fall Prior Risk Assmnt	8555	11	1039	7	12.1%	-	-		-	-	

Note: *N*_0_: Total number of units with data.

*N*_1_: Total number of units with indicator value changed after data cleaning.

*N*_2_: Total number of units identified by each method for potential outlier check.

*N*_3_: Total number of units with indicator value checked and corrected (or dropped).

*: Denote the method failed.

Post: False alarm rates for post clean data.

### Monte Carlo simulation

The Gamma distribution is a general type of distribution ranging from nearly symmetric normal to extremely skewed exponential distributions. The skewness of a Gamma variable can be fully described with a shape parameter. We chose Gamma random variables to imitate the highly skewed NDNQI indicators, which were constructed in order to pinpoint rare but inadequate supply of Total Nursing Care Hours Per Patient Day (Table 1). The SAS Gamma random number generating function RANGAM was used with different sets of seed, shape (*α*), and scale (*β*) parameters to generate a data set of 1,000 observations at each *α* by *β* combination. We let *β* to vary from 2 to 18, such that *α* from 4 to 4/9, in order to maintain the same mean ( μ=α×β) of 8.00 for all data sets but with varying degrees in skewness from 0 to 4. Potential outliers for each generated data set are identified at the 1.25^th^ and 98.75^th^ percentile levels by all three methods. With each set of shape parameter *α*, the skewness is calculated as $y=\frac{2}{\sqrt{\alpha }}$. We then calculated the proportion of potential outliers for each data set by FAST-MCD, MAD, and IQR approach, and summarize for each method by the level of γ with the mean and standard deviation of the proportion of potential outliers. The estimated skewness for each data set was obtained with SAS UNIVARIATE procedure (Table 3).

**Table 3:** False alarm rate as a function of skewness in data distribution for IQR, MAD, or FAST-MCD approach with simulation

**Asymmetry in data distribution**		**Potential outlier rate by different methods**			
**Preset skewness**	**Estimated skewness**	**Percentile**	**IQR**	**MAD**	**FAST-MCD**
0.000	−0.01(0.077)	0.025(0.005)	0.026(0.007)	0.023(0.007)	0.027(0.008)
1.000	0.983(0.116)	0.025(0.005)	0.035(0.006)	0.036(0.006)	0.078(0.013)
1.414	1.398(0.174)	0.025(0.005)	0.045(0.006)	0.049(0.007)	0.129(0.015)
1.732	1.720(0.221)	0.025(0.005)	0.053(0.007)	0.062(0.007)	0.186(0.014)
2.000	1.959(0.249)	0.025(0.005)	0.060(0.007)	0.073(0.008)	0.227(0.014)
2.236	2.197(0.286)	0.025(0.005)	0.066(0.007)	0.085(0.008)	0.260(0.014)
2.449	2.397(0.318)	0.025(0.005)	0.072(0.007)	0.097(0.009)	0.288(0.014)
2.646	2.581(0.343)	0.025(0.005)	0.077(0.007)	0.108(0.009)	0.313(0.014)
2.828	2.759(0.375)	0.025(0.005)	0.082(0.007)	0.120(0.009)	0.333(0.013)
3.000	2.928(0.409)	0.025(0.005)	0.087(0.007)	0.132(0.009)	0.351(0.013)
3.162	3.076(0.433)	0.025(0.005)	0.092(0.007)	0.144(0.019)	0.367(0.013)
3.317	3.225(0.467)	0.025(0.005)	0.096(0.007)	0.194(0.101)	0.380(0.012)
3.464	3.364(0.502)	0.025(0.005)	0.099(0.007)	0.388(0.167)	0.392(0.011)

Mean rate of potential outliers with standard deviation in parenthesis for 1,000 simulated data sets at each preset skewness level.

**Figure 2:**
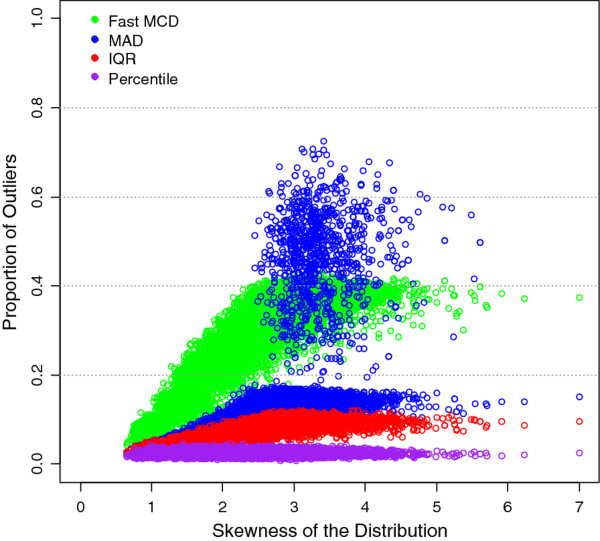
False alarm rate for potential outliers varies greatly with different approaches if data is highly skewed in distribution, but remain close to each other if skewness is close to zero.

With skewness increasing from 1.00 to around 3.00, all three approaches tend to over-estimate the false alarm rate than the targeted 2.5% significance level, but the magnitude is quite different. With IQR approach, the overestimate ranges from 0.1% at γ = 1.00 to 5.1% at γ = 3.00, in contrast to from 0.3% to 9.7% or from 2.8% to 30.1% for MAD or FAST-MCD approaches, respectively. This indicates that, 1) the FAST-MCD, IQR, and MAD methods are within the range of natural variation from the 2.5% target and approach each other only when the data are approximately normally distributed (γ = 0); 2) FAST-MCD could inflate the false alarm rates as high as 30% in contrast to 5% for the IQR and 10% for MAD approaches if the data is highly skewed to the higher end (γ = 3.00). On average, the robustness to asymmetry in data distribution is ordered by IQR > MAD > FAST-MCD (Figure 2). However, the behavior of the MAD approach is erratic (if γ >2) as reflected by quite a few cases with larger than usual estimates of proportion for potential outliers over the target (Figure 2) and the large variations in proportion (Table 3).

Outliers differ from extreme values of the same distribution. To examine the ability to pick up true outliers by each method, we insert 1% observations from *N* (60, 9/4) that differ from the remaining 99%. Again, the bulk of the data (990 out of 1,000) is generated with different shape and scale parameter with Gamma distribution (μ = 8.00). We set the skewness at 1.00, 2.00, and 3.00, corresponding to a variance (σ^2^) of $16\times \sqrt{2},8\sqrt{2},\text{and}\;16/3\times \sqrt{2}\;$, respectively. At different skewness, we compare the three methods and see if the inserted true outliers are picked up and whether the overall proportion of potential outliers by each method approaches to the targeted 5% level (Table 4). All three methods were able to identify 10 out of 10 (100%) of the planted outliers regardless the severity of skewness in data distribution. Extreme values, along with the planted known outliers, could be identified as false outliers at a much higher rate by the FAST-MCD or MAD than the IQR approach when the bulk of the data is skewed to the right (γ >1). The rate of false outliers reach as high as 30%, 15%, and 10% at γ =3.00 for FAST-MCD, MAD, IQR, and 20%, 8%, and 7% at γ =2.00, but are barely distinguishable between MAD and IQR, and only slightly higher for FAST-MCD approach at γ =1.00 (Table 4).

**Table 4:** Skewness in data distribution inflate overall false alarm rate with the presence of true outliers but with different scale depending on whether IQR, MAD, or FAST-MCD approach is used

**Asymmetry in data distribution**	**Data composition**		**True and false outlier rates by different approach**		
**Preset skewness**	**Planted outliers**	**Simulated observations**	**IQR**	**MAD**	**FAST-MCD**
0.000	10		1.000(0.000)	1.000(0.000)	1.000(0.000)
1.000		990	0.028(0.006)	0.029(0.006)	0.076(0.013)
Overall Outliers Detected / (10 + 990)			0.038	0.039	0.085
0.000	10		1.000(0.000)	1.000(0.000)	1.000(0.000)
2.000		990	0.053(0.006)	0.066(0.008)	0.223(0.014)
Overall Outliers Detected / (10 + 990)			0.062	0.075	0.231
0.000	10		1.000(0.000)	1.000(0.000)	1.000(0.000)
3.000		990	0.081(0.006)	0.123(0.008)	0.344(0.012)
Overall Outliers Detected / (10 + 990)			0.090	0.131	0.351

Planted outliers from normal distribution, and simulated observations from Gamma distribution.

## Conclusion and discussion

When used for one dimensional outlier detection in raw data, the robustness and efficiency of the ad-hoc, distance-based IQR and MAD, as well as the classic theoretically based FAST-MCD approaches depends on the skewness in data distribution. Most previous studies focused on regression residuals with the majority of the observations being normally distributed or relatively symmetric, a precondition that makes the FAST-MCD robust (free from masking and swarming) and sensitive to the presence of multiple outliers. With Monte Carlo simulation and NDNQI examples, we demonstrated that, with skewed data and preselected critical value, the FAST-MCD approach could be misleading by overestimating false alarm rate than the targeted level. Consequently, it was less efficient because more time and resources need to be committed to find the true, among all flagged, potential outliers at the same significance levels, compared to the IQR or MAD approaches. Notice, a limitation to the MAD and FAST-MCD is with the application to 0-inflated data. As many NDNQI indicators reflect rare adverse events, a median value of 0 is not uncommon, causing both methods to fail. In certain indicator distributions, even the IQR method has limitations as the 75^th^ percentile is 0.

The primary goal for initial input data screening with large database is to achieve high data quality with less time and effort. It can be argued that, without constraints in time and effort, one can always achieve higher quality by duplicating data entries, double checking every observation, or relaxing the significance level for the false alarm rate with any method. Winskowski et al. [34] reported, for example, that the detection capability was increased by increasing the significance level of *α* from 0.05 to 0.20 without severe impact to false alarm probabilities for the randomly scattered outliers in the interior of the *X*-space. While this may be true for small datasets with low contamination and plausible to limited number of variables, a key question for extensive data based research is how to maintain balance between data quality control and limits and constraints in time and resources. At NDNQI, we strive to deliver quarterly reports to member hospitals within three weeks after a quarterly data entry was over. Unlike residual from regression analysis, on the other hand, most statistical data screening for quality control deals with raw data whose distribution may be anything but normal in nature. Over estimating the false alarm rates for potential outliers, could dramatically reduce the efficiency and add extra burden for data entry at hospital sites and database management at NDNQI administration. Instead of FAST-MCD, the IQR or MAD approach can be used to maintain the targeted significance level for potential outlier check without suffering a substantial loss in sensitivity for the presence of true outliers and a dramatic increase in false alarm rate. Notice that the critical-value based approach we currently used may not be most optimal considering the quantity of univariate measures checked for outliers, as recent literature suggested that a data dependent choice of critical-vale for the FAST-MCD approach can achieve full efficiency and control the false alarm rates [10].

Real case application with 2007 NDNQI 4^th^ quarter data indicated that as much as 20% more observations need not to be checked with FAST-MCD (6 times more) than with IQR or MAD to achieve the goal of screening the same sets of true outliers (Table 2). However, erratic behavior can be expected with MAD approach (Figure 2), in some cases worse than FAST-MCD (e.g., Assault Rate).

Most statistics for detecting outliers suffer from masking effect as a result of inflation in scale estimates when multiple outliers are present. FAST-MCD avoids masking by assigning zero weight to every outlier, while IQR and MAD are generally robust to such effect by using ordered statistics. However, neither IQR nor MAD approach should be regarded as free from distributional effect because using ordered statistics for estimating scale does not change the fact that the extreme observations still lead to biased estimates for location. As a result, both IQR and MAD approach can not avoid masking and swarming effect for data with high rate of contamination. For example, if *m* contaminated true outliers hide in *n* total observations, the property of IQR and MAD may depend on the scale and proportions of the *m* outliers since the ordered statistics may shift to one of the *m* outliers from that of the (*n*-*m*) uncontaminated observations if the target population is highly contaminated.

Data transformation provides a powerful tool for developing a parsimonious model when the variable of interest deviates from normal in distribution [5]. Applying the FAST-MCD approach on a transformed scale can be useful to detect potential outliers without inflating the false alarm rate but is beyond the scope of this paper. In multivariate analysis, FAST-MCD approach remains to be most popular and feasible for outlier check with data in multiple dimensions, but how asymmetry in data distribution affect the robustness in multivariate case need further investigation.

## Competing interests

The first author, Qingjiang Hou, who is now working at Cerner Corporation, along with co-authors Brandon Crosser, Jonathan D Mahnken, Byron J Gajewski, and Nancy Dunton, who have been working at The Kansas University Medical Center, declare that they have no competing interests.

## Authors' contributions

QH developed the statistical analysis framework, conducted literature review, and prepared the manuscript; BC reviewed and edited the manuscript in the context of current NDNQI quarterly report processing and updated the manuscript/analysis accordingly; JM and BG provided methodological guidance on statistical analysis and provided critical input to the manuscript. ND supervised NDNQI data collection, evaluated unit-specific nurse-sensitive data, and provided overall guidance for the manuscript. All authors read and approved the final manuscript. QH was employed by Kansas University Medical Center at the time of the research and is currently a Scientist/Biostatistician with Cerner Corporation ( http://www.cerner.com).
